# The Change of Fingolimod Patient Profiles over Time: A Descriptive Analysis of Two Non-Interventional Studies PANGAEA and PANGAEA 2.0

**DOI:** 10.3390/jpm11060561

**Published:** 2021-06-16

**Authors:** Tjalf Ziemssen, Ulf Schulze-Topphoff

**Affiliations:** 1 Zentrum für Klinische Neurowissenschaften, Universitätsklinikum Carl Gustav Carus, D-01307 Dresden, Germany; 2 Novartis Pharma GmbH, D-90429 Nuremberg, Germany; ulf.schulze_topphoff@novartis.com

**Keywords:** fingolimod, real-world evidence, patient profiles

## Abstract

(1) Background: Fingolimod (Gilenya^®^) was the first oral treatment for patients with relapsing-remitting multiple sclerosis (RRMS). Since its approval, the treatment landscape has changed enormously. (2) Methods: Data of PANGAEA and PANGAEA 2.0, two German real-world studies, were descriptively analysed for possible evolution of patient profiles and treatment behavior. Both are prospective, multi-center, non-interventional, long-term studies on fingolimod use in RRMS in real life. Data of 4229 PANGAEA patients (recruited 2011–2013) and 2441 PANGAEA 2.0 patients (recruited 2015–2018) were available. Baseline data included demographics, RRMS characteristics and disease severity. (3) Results: The mean age of PANGAEA and PANGAEA 2.0 patients was similar (38.8 vs. 39.2 years). Patients in PANGAEA 2.0 had shorter disease duration (7.1 vs. 8.2 years) and fewer relapses in the year before baseline (1.2 vs. 1.6). Disease severity at baseline estimated by EDSS and SDMT was lower in PANGAEA 2.0 patients compared to PANGAEA (EDSS difference 1.0 points; SDMT difference 3.3 points). (4) Conclusions: The results hint at an influence of changes in the treatment guidelines and the label on fingolimod patients profiles over time. Patients tended to have lower disease activity at fingolimod initiation, suggesting an earlier intervention. This indicates increased experience in using fingolimod for sub-optimally treated RRMS patients and a change in mindset towards an early treatment optimization.

## 1. Introduction

Fingolimod (Gilenya^®^, Novartis AG) was first approved in 2011 as a once-daily oral treatment for patients with highly active relapsing-remitting multiple sclerosis (RRMS). Since its approval, the treatment landscape has changed enormously and the label wording of fingolimod has been adapted several times, which might have altered the profile of patients starting fingolimod treatment. Two large real-world studies were conducted in Germany, which could give insight into how patient characteristics might have changed over time.

**Figure 1:**
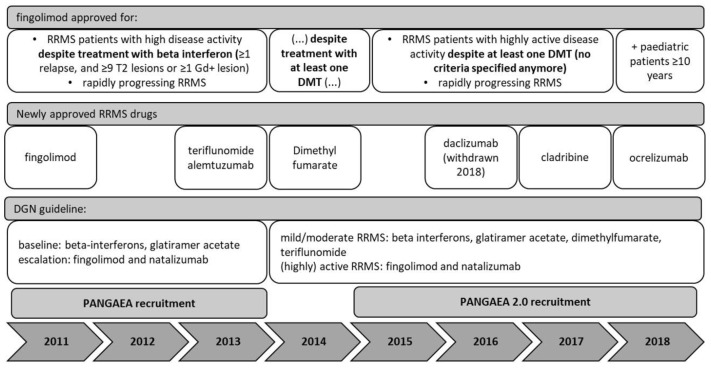
Changes in the treatment landscape during recruitment into PANGAEA and PANGAEA 2.0.

The PANGAEA study recruited patients from 2011 to 2013. At that time, fingolimod was approved for use in RRMS patients fulfilling specific disease activity criteria despite beta interferon pretreatment and in rapidly progressing RRMS. The German guidelines published by the DGN (German Society for Neurology) recommended beta interferons and glatiramer acetate for first-line RRMS treatment as well as fingolimod and natalizumab for escalation therapy. Second-line options were azathioprine, intravenous immunoglobulin, mitoxantrone or cyclophosphamide, which are rather unspecific immunosuppressive or cytotoxic drugs mainly used in oncology or organ transplantation settings. In 2013, teriflunomide and alemtuzumab emerged as new treatment alternatives for RRMS patients (Figure 1).

The consecutive real-world study PANGAEA 2.0 recruited patients from 2015 to 2018. By this time, the label of fingolimod had already been extended to the use in patients with disease activity despite any DMT and without the need to fulfil specific activity criteria. An updated version of the DGN guideline published in 2014 no longer distinguishes between baseline and escalation therapy, but classifies treatments by their efficacy for use in mild/moderate and in (highly) active forms of RRMS. Beta interferons, glatiramer acetate, dimethylfumarate and teriflunomide have since been recommended as first-line treatment options for mild/moderate RRMS, while fingolimod, alemtuzumab, and natalizumab are recommended as first-line treatments for (highly) active RRMS [1]. Furthermore, new MS therapies have gained approval, i.e., dimethylfumarate (2014), daclizumab (2016, withdrawn 2018 due to safety concerns), cladribine (2017) and ocrelizumab (2018) (Figure 1).

In this analysis, we compared baseline data from PANGAEA and PANGAEA 2.0 in order to evaluate the change in the clinical profile of RRMS patients who switched to fingolimod between 2011–2013 (PANGAEA) and 2015–2018 (PANGAEA 2.0) [2,3].

## 2. Materials and Methods

PANGAEA 2.0 and PANGAEA were prospective, multi-center, non-interventional, long-term studies of fingolimod (0.5 mg) for the treatment of patients with RRMS. The studies were conducted in Germany including office-based neurologists and neurology clinics. Patients who received fingolimod according to the summary of product characteristics were eligible. Treatment followed common clinical routine. The observation period was up to 36 months in PANGAEA 2.0 and up to 60 months in PANGAEA. Follow-up visits were documented about every three months. While PANGAEA 2.0 only included patients naive to fingolimod treatment with a focus on patients with disease activity (i.e., relapses, MRI activity) despite current treatment with any DMT, PANGAEA also recruited patients who had already been treated with fingolimod in a clinical study setting before. For the purpose of this analysis, only data of PANGAEA patients treated with fingolimod for the first time were considered to allow for comparison between both study populations.

This post hoc analysis of the data of PANGAEA 2.0 and PANGAEA was conducted to gain insight on the clinical profile of patients initiating fingolimod nowadays and patients who started fingolimod in the first years after marketing approval. Therefore, baseline data of RRMS patients who started on fingolimod during the years 2011 to 2013 in PANGAEA and baseline data of patients who initiated treatment between 2015 to 2018 in PANGAEA 2.0 were descriptively analysed.

Before 2011, treatment alternatives for patients not responding on beta interferons or glatiramer acetate were very limited. Due to this lack of effective treatment for highly active disease, patients usually continued on their baseline medication for years despite signs for inadequate control of the disease. At the beginning of PANGAEA recruitment, patients with a longer disease duration or more severe disease therefore might be overrepresented. In order to exclude patients with a potential for more severe disease conditions and longer disease duration due to a lack of treatment alternatives before 2011, a sensitivity analysis of PANGAEA was performed. For this analysis, only patients recruited in 2012 and 2013 were considered.

Baseline data included demographic variables (sex, age, body mass index), MS characteristics (disease duration, number of relapses in the past year), disease severity as measured by the expanded disability status scale (EDSS), clinical global impression (CGI) severity, and the multiple sclerosis severity score (MSSS), as well as cognitive impairment as measured by the symbol digit modalities test (SDMT).

The studies were conducted in compliance with the principles of the Declaration of Helsinki and according to the current recommendations for observational studies in Germany of the following institutions: the Voluntary Self-control of Pharmaceutical Companies (FSA) codex, the Federal Institute for Drugs and Medical Devices, the Paul-Ehrlich-Institut (PEI), and the Research-Based Pharmaceutical Companies (vfa). The studies are registered in the vfa database for non-interventional studies. Prior to study initiation, an ethics committee was consulted, and the study was mentioned to the competent higher federal authority, the Federal Association of Statutory Health Insurance Physicians, and the Statutory Health Insurance. Patients were only included after providing written informed consent at the time of the baseline visit.

Presented data are part of analyses conducted in January 2018 (PANGAEA) and October 2019 (PANGAEA 2.0). All data were analysed descriptively using SAS version 9.2. Demographic baseline characteristics and disease history were analysed for the total population of each study. Continuous data were analysed as mean and standard deviation, categorical data as absolute and relative frequencies. All data were analysed as observed, and missing values were not imputed.

## 3. Results

In total, 2578 patients from 250 sites were recruited in PANGAEA 2.0 during June 2015 and March 2019. The present analysis includes 2441 PANGAEA 2.0 patients. Recruitment into PANGAEA started in May 2011 and was finished in December 2013 with 4229 enrolled patients from 374 sites. Of these patients, 3188 were fingolimod-naive at the time of enrolment. Only these patients were considered for the present analysis, as PANGAEA 2.0 only recruited patients naive to fingolimod. For the sensitivity analysis, 2152 patients recruited between 2012 and 2013 were investigated.

The mean age of PANGAEA and PANGAEA 2.0 patients was similar (38.8 ± 10.1 vs. 39.2 ± 10.8 years, mean ± SD) (Table 1). The proportion of patients younger than 30 years and older than 50 years was higher in PANGAEA 2.0 (25.2% vs. 23.1%) and (16.9% vs. 12.0%). In PANGAEA, more patients at the age from 30 to 50 years were included in comparison to PANGAEA 2.0 (64.9% vs. 57.9%; data not shown).

**Figure 2:**
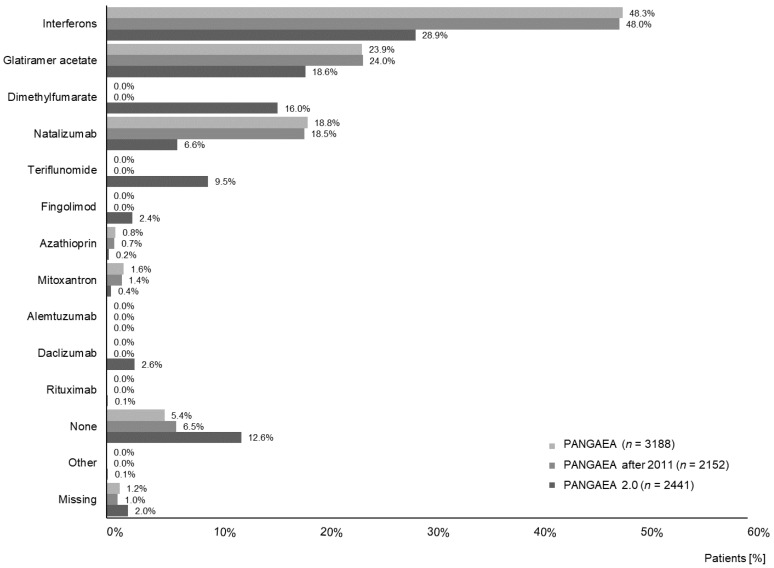
Previous disease-modifying therapy.

In PANGAEA 2.0, less patients previously treated with beta interferons, glatiramer acetate, and natalizumab were enrolled compared to PANGAEA (54.1% vs. 91.0%). In addition, 28.1% of PANGAEA 2.0 patients had received dimethylfumarate, teriflunomide, alemtuzumab or daclizumab before enrolment. All of the aforementioned drugs had not yet been available at the time of PANGAEA enrolment (Figure 2).

**Figure 3:**
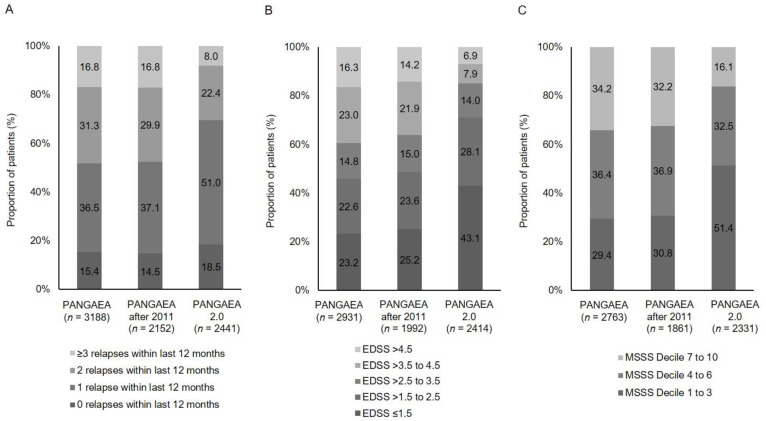
Number of relapses (**A**); EDSS (**B**); and MSSS (**C**) by category at baseline (EDSS, Expanded Disability Status Scale, MSSS, Multiple Sclerosis Severity Scale).

Patients in PANGAEA 2.0 had a shorter disease history (7.1 ± 6.5 vs. 8.2 ± 6.4, mean ± SD) and fewer relapses in the last 12 months before baseline compared to PANGAEA (1.2 ± 1.0 vs. 1.6 ± 1.2, mean ± SD) (Table 2). More patients in PANGAEA 2.0 had none or only one relapse in the last 12 months before baseline (69.5% vs. 51.9%). In contrast, more patients in PANGAEA experienced two or more relapses in the year before baseline (48.1% vs. 30.4%) (Figure 3).

**Figure 4:**
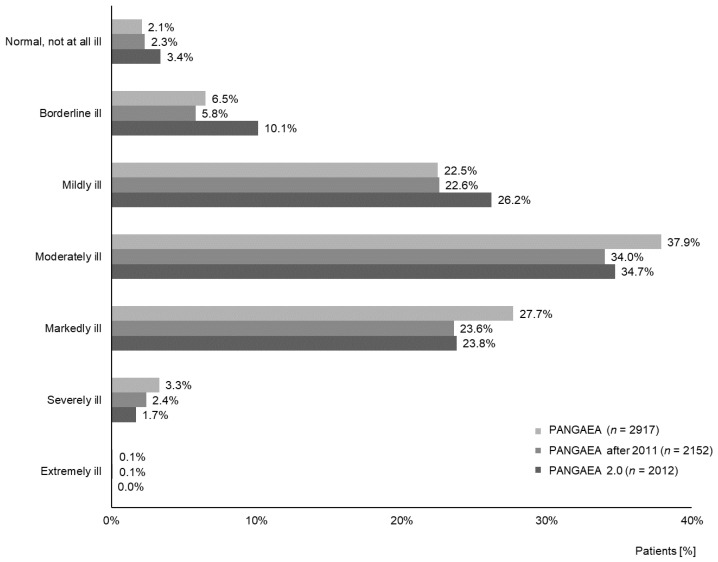
CGI severity of illness at baseline (CGI, Clinical Global Impression).

Disease severity at baseline was lower in PANGAEA 2.0 patients in comparison to PANGAEA. Accordingly, mean EDSS in PANGAEA 2.0 was 2.1 ± 1.6 vs. 3.1 ± 1.7 in PANGAEA (Table 2). In PANGAEA 2.0, 85.2% had a baseline EDSS of 3.5 or lower vs. 60.6% in PANGAEA, i.e., a 1.4-fold increase in proportion. On the other hand, 14.8% had a baseline EDSS of 4 or higher in PANGAEA 2.0 vs. 39.3% in PANGAEA (Figure 3). In addition, 51.4% of the patients in PANGAEA 2.0 had an MSSS at baseline in deciles 1 to 3 compared to 29.4% in PANGAEA. Conversely, 34.2% of the patients in PANGAEA had an MSSS in the deciles 7 to 10 compared to 16.1% in PANGAEA 2.0 (Figure 3). CGI severity of illness at baseline shows that a higher proportion of patients in PANGAEA 2.0 were not at all ill, borderline ill or mildly ill at baseline compared to PANGAEA (39.7% vs. 31.1%). In PANGAEA, more patients were moderately, markedly, severely or extremely ill compared to PANGAEA 2.0 (69.0% vs. 60.2%; Figure 4). The mean SDMT score was higher in PANGAEA 2.0 compared to PANGAEA with a mean difference of 3.3 points (Table 2).

Data of the sensitivity analysis of PANGAEA (patients recruited in 2012 and 2013) are in line with data of the total PANGAEA analysis. No differences in demographics and disease characteristics were identified (Table 1 and Table 2; Figure 2, Figure 3 and Figure 4).

## 4. Discussion

The two German non-interventional studies PANGAEA and PANGAEA 2.0, which were conducted to gather information on the effectiveness and safety of fingolimod in daily practice, are the largest studies to collect real-world data on patients with highly active RRMS in Germany. Over 6000 patients were included between 2011 and 2018, a period during which the MS treatment landscape changed dramatically. As in the German health care system, the use of MS drugs is not impacted by cost restraints and any approved MS drug can therefore be prescribed, real-world data from Germany presumably maps the change in the treatment landscape. Data of PANGAEA and PANGAEA 2.0 therefore are of unique value for an analysis of how patient characteristics might have changed by the evolving MS market. Therefore, the demographic and disease characteristic profiles of RRMS patients initiated on treatment with fingolimod in both studies have been analysed. It was of particular interest whether the patient profiles indicate an earlier adaption of therapeutic intervention in highly active RRMS patients after new treatments became available and recommendations as well as the fingolimod label has been changed towards a greater flexibility for therapy optimization.

The analysis suggests that patients included into PANGAEA 2.0 (2015–2018) switched to fingolimod at an earlier stage of disease with respect to disease characteristics and impairment status in comparison to PANGAEA (2011–2013). In detail, patients had a shorter disease duration, fewer relapses in the past year, and a lower disease severity according to EDSS, CGI severity and MSSS. The difference in the mean EDSS between the study populations of 1.0 indicates possibly relevant changes, as disability progression is defined as a 1.0-point increase (if EDSS score is <5.5), and as a 0.5-point increase (if EDSS score is ≥5.5) [4,5]. Furthermore, the proportion of patients switching to fingolimod at an EDSS below 4.0 increased by 40%. At an EDSS below 4.0, patients show only mild to moderate walking impairment, i.e., they are still able to walk more than 500 m without aid and rest [6]. The present data therefore indicate that RRMS treatment was optimized earlier during the observational period of 2015–2018, not only at a later stage, when patients are already markedly impaired.

This assumption not only applies to the level of motor impairments, but also to cognitive function. The extent of cognitive impairment in PANGAEA 2.0 patients seems to be lower. Recent research supports a responder definition of SDMT change of approximately four points or 10% in magnitude, although it is not established as a between group minimal important difference [7]. Others have reported that patients with evidenced deterioration in vocational status had a decline of three points in the SDMT [8]. With respect to the large group size analysed and the observed between group difference of 3.4 points, a trend towards a lower level of cognitive impairments at the time of treatment change can be assumed, indicating that cognition might likewise be affected by changes in treatment paradigms. However, the observations regarding disability status and cognitive impairment need further confirmation.

Undelayed treatment optimization is of very high importance for the long-term outcome. A post hoc analysis of the pivotal FREEDOM and FREEDOM II trials of fingolimod has shown that an early intervention is important to protect against disease-related disability in the long term. Accordingly, immediate fingolimod treatment vs. delayed treatment was associated with a significant reduction of the annual relapse rate by 45% and a significant reduction in the risk for confirmed disability progression by 24%. The risk of reaching an EDSS ≥4 was significantly reduced by 32%. The effect was even more pronounced in younger patients. Moreover, in young adults, the analysis was able to show even significant confirmed disability improvement when immediately treated [9]. Patients escalated from glatiramer acetate or beta interferon to fingolimod, alemtuzumab or natalizumab within five years of disease onset compared to patients with a later treatment switch had a 24% lower probability of conversion to a secondary progressive disease course [10]. The more pronounced effect in younger patients can be explained by neurological reserve, i.e., the brain’s capacity to compensate for and repair damaged tissue. At some point of the disease course, the neurological reserve will be exhausted and the brain cannot compensate the damage done by inflammatory processes anymore. Using effective DMT treatment delays irreversible damage to the brain [11]. Consequently, the better and the earlier inflammatory activity is suppressed in these patients, the longer the neurological capacities can be protected, leading to a delay of irreversible disability progression.

The possible difference in patient characteristics indicated in this analysis might have been driven by recruitment of patients with more severe diseases at the beginning of PANGAEA in 2011. During the years before approval of fingolimod, no or few treatment alternatives for a highly active disease were available. Thus, patients might have had a more advanced disease. To investigate this possibility, a sensitivity analysis of PANGAEA was applied, excluding patients recruited during the early phase of fingolimod availability in 2011. The results of this additional analysis were all in line with the data of the total PANGAEA analysis, which rules out that the observed differences were confounded. Overall, a change to earlier optimization of sub-optimally treated patients with RRMS in Germany over time (2011–2018) can be assumed.

The possible change of patient profiles indicated here might therefore be a direct consequence of the omission of detailed disease activity criteria in the label of fingolimod, which enables more individualized treatment decisions and an earlier intervention in case of inadequate treatment. Alongside with the modification of these formal conditions over the years, the growing experience gained with fingolimod in the past years might have additionally impacted treatment decisions. Fingolimod has been reported to be safe in the long term [12]. This increases confidence in the drug and its prompt use in patients with high disease activity. It might also help to broaden its use to special patient populations in need of an effective therapy, e.g., younger or older patients.

Furthermore, the changing landscape in RRMS treatment options might have influenced patient profiles. This can be assumed as less patients were previously treated with beta interferons, glatiramer acetate, and natalizumab in PANGAEA 2.0 compared to PANGAEA. A relevant proportion of patients had received newer drugs, i.e., dimethylfumarate or teriflunomide, which are also recommended as first-line agents for mild/moderate RRMS, before switching to fingolimod [1]. Almost 20% of the patients had received natalizumab before entering PANGAEA, almost three times as many as in PANGAEA 2.0. Before the approval of fingolimod, natalizumab was the only guideline recommended DMT for patients with highly active disease. However, its safety profile limits the duration of treatment and the availability of fingolimod might have altered the benefit–risk assessment of prolonged natalizumab treatment towards a switch to fingolimod. Previous analyses of PANGAEA data suggest that the use of fingolimod after natalizumab has favorable effectiveness and tolerability over 48 months. In total, 58.2% of the previous natalizumab patients remained on fingolimod over this period, the annualized relapse rate was approximately 0.5 in this population and only 17.1% had 6-month confirmed disability worsening [13].

With respect to patients who came from DMT options for mild and moderate disease before fingolimod was initiated, it remains unclear, from the present analysis, whether they were cycled within this DMT group before or whether they had received a highly effective therapy as first intervention. According to a non-interventional study from Germany with 595 patients, more than half of the patients with disease activity despite DMT (54.3%) switched between DMTs for mild and moderate RRMS, and only 43.5% received treatment for a highly active disease. The authors concluded that DMT usage is still suboptimal [14]. This is in line with data from a survey among MS patients in Germany. In the survey, 38.9% of these patients had experienced one or more relapses, two thirds of the patients with relapses were under immunomodulatory treatment, leaving approximately one third untreated patients [15]. These data indicate that there is still a huge potential for improvement in order to prevent long-term disability progression and conversion to the progressive phase of the disease. Early intervention with highly active drugs is the most promising and already available tool to achieve this goal [10,16]. Interestingly, patients in both studies, PANGAEA and PANGAEA 2.0, had a similar mean age of 39 years with no trend towards earlier treatment initiation with respect to age. Given that fingolimod is efficacious and safe to use in the treatment of young adults [17], there is still much room for improvement regarding earlier intervention in case of disease activity.

Nevertheless, the trend for lower disease severity in patients with more recent fingolimod initiation suggests that disease management has already improved to some extent and that treatments are switched earlier in an endeavor of finding the patient individual adequate treatment to improve long-term outcomes.

## 5. Conclusions

In summary, various new treatment options, the revised treatment guidelines and especially the label changes seem to have influenced the clinical profiles of fingolimod patients over time. The present analysis hints at a lower disease activity at the time of fingolimod initiation, suggesting earlier intervention in case of inadequately controlled disease. This indicates an increased experience in using fingolimod for sub-optimally treated RRMS patients and a change in mindset towards an early treatment optimization to improve long-term outcome. Due to the descriptive character of the analysis, the present assumptions need further investigation in a confirmative setting.

## Figures and Tables

**Table 1:** Demographics.

	PANGAEA *n* = 3188	PANGAEA (Recruited after 2011); *n* = 2152	PANGAEA 2.0 *n* = 2441
Female, *n* (%)	2284 (71.7)	1542 (71.7)	1740 (71.3)
Age, Mean ± SD	38.8 ± 10.1	38.6 ± 10.3	39.2 ± 10.8
BMI, Mean ± SD	25.2 ± 5.2	25.4 ± 5.4	25.7 ± 5.4

BMI, Body Mass Index; *n*, number of patients in the total analysis population; SD, Standard deviation.

**Table 2:** Disease characteristics.

	PANGAEA *n* = 3188	PANGAEA (Recruited after 2011); *n* = 2152	PANGAEA 2.0 *n* = 2441
MS duration since diagnosis in years, Mean ± SD	8.2 ± 6.4	7.9 ± 6.4	7.1 ± 6.5
Number of MS relapses within last 12 months, Mean ± SD	1.6 ± 1.2	1.6 ± 1.1	1.2 ± 1.0
Number of MS relapses within last 24 months, Mean ± SD	2.4 ± 1.7	2.3 ± 1.7	1.4 ± 1.3
EDSS, Mean ± SD	3.1 ± 1.7	3.0 ± 1.7	2.1 ± 1.6
SDMT score, Mean ± SD	47.1 ± 13.7	48.8 ± 12.9	50.4 ± 15.9

EDSS, Expanded Disability Status Scale; MS, Multiple sclerosis; *n*, number of patients in the total analysis population; SD, Standard deviation; SDMT, Symbol Digit Modalities Test.

## Data Availability

The data presented in this study are available on request from the corresponding author. The data are not publicly available due to data privacy reasons.
